# Buildings Contemplated

**Published:** 1914-05-23

**Authors:** 


### Buildings Contemplated.

Brigg.—The Guardians have received the approval of the Local Government Board to the plans for a new workhouse infirmary.

Bromley.—The Bromley (Kent) Guardians have approved the preliminary plans for the proposed new infirmary.

Clayton.—The Wakefield Corporation have passed plans for alterations and additions to the Clayton Hospital.

Duffield.—The East Riding County Council have ordered the preparation of plans for an isolation hospital. The cost is estimated at £10,935.

Leytonstone.—The West Ham Guardians invite tenders for the erection and completion of an $x$-ray room at the infirmary. Forms of tender may be obtained from the Clerk, Union Road, Leytonstone, N.E.

Llanelly.—Plans for the additions to the workhouse infirmary have been referred to the house committee of the Guardians.

London.—The London Jewish Hospital Association hope to start building in a few weeks a new hospital which will cost over £10,000.

Ripon.—Tenders are invited for the alteration and additions to the Ripon Cottage Hospital. Messrs. Bown and Crosland, Harrogate, are the architects, from whom quantities may be obtained.

Skipton.—The Rural District Council have requested the surveyor to prepare plans and estimates for a smallpox hospital on the Sandy Beck Bar site.

Wakefield.—The Corporation have applied to the Local Government Board for sanction to borrow £13,850 for the extension of the infectious diseases hospital.

